# Transcranial Electrical Currents to Probe EEG Brain Rhythms and Memory Consolidation during Sleep in Humans

**DOI:** 10.1371/journal.pone.0016905

**Published:** 2011-02-14

**Authors:** Lisa Marshall, Roumen Kirov, Julian Brade, Matthias Mölle, Jan Born

**Affiliations:** 1 Department of Neuroendocrinology, University of Lübeck, Lübeck, Germany; 2 Institute of Neurobiology, Bulgarian Academy of Sciences, Sofia, Bulgaria; 3 Institute of Medical Psychology and Behavioral Neurobiology, University of Tübingen, Tübingen, Germany; University of Alberta, Canada

## Abstract

Previously the application of a weak electric anodal current oscillating with a frequency of the sleep slow oscillation (∼0.75 Hz) during non-rapid eye movement sleep (NonREM) sleep boosted endogenous slow oscillation activity and enhanced sleep-associated memory consolidation. The slow oscillations occurring during NonREM sleep and theta oscillations present during REM sleep have been considered of critical relevance for memory formation. Here transcranial direct current stimulation (tDCS) oscillating at 5 Hz, i.e., within the theta frequency range (theta-tDCS) is applied during NonREM and REM sleep. Theta-tDCS during NonREM sleep produced a global decrease in slow oscillatory activity conjoint with a local reduction of frontal slow EEG spindle power (8–12 Hz) and a decrement in consolidation of declarative memory, underlining the relevance of these cortical oscillations for sleep-dependent memory consolidation. In contrast, during REM sleep theta-tDCS appears to increase global gamma (25–45 Hz) activity, indicating a clear brain state-dependency of theta-tDCS. More generally, results demonstrate the suitability of oscillating-tDCS as a tool to analyze functions of endogenous EEG rhythms and underlying endogenous electric fields as well as the interactions between EEG rhythms of different frequencies.

## Introduction

An increasing number of studies are investigating the impact of applied oscillatory electric currents or fields, inducing or estimated to induce subthreshold membrane potential oscillations, on the activity of cortical networks and/or individual neurons as well as their functional implications [1]–[8]. We have focused on the functional implications of applied currents during sleep on sleep-dependent memory consolidation [2], [9] as well as on the state dependence of these effects [5]. There is compelling evidence that the distinct stages of sleep play an essential role in the long-term consolidation of memories [10], [11]. Specifically, slow-wave sleep (SWS), which is hallmarked by slow oscillatory activity (<1 Hz) in the human electro-encephalogram (EEG) and is most pronounced during the first part of nocturnal sleep, has been implicated in the consolidation of declarative memories. Weak electric currents oscillating within the frequency range of the sleep slow oscillation in humans (∼0.75 Hz) and an anodal DC bias applied at the transition into SWS enhanced EEG power of the slow oscillation and sleep spindle activity as well as declarative memory consolidation [2].

The consolidation of various procedural tasks appears to benefit more from sleep during the later part of the night which is dominated by prolonged periods of rapid eye movement (REM) sleep, but also by lighter stage 2 NonREM sleep [12], [13]. Results on the effect of REM sleep and REM sleep specific EEG brain rhythms on memory consolidation are inconsistent [11], [14]. Theta oscillations are a hallmark of the EEG during REM sleep in rodents [15], [16] and are also characteristic for REM sleep in humans, though in a more transient form [17]–[19].

The aim of the present experiments both applying an anodal current oscillating at theta frequency (theta-tDCS) during either Non-REM or REM sleep is to determine firstly, the impact of ongoing brain electric activity and brain state on the ability to modulate EEG activity by weak electric currents, and secondly to determine the functional implication on memory consolidation. We find that effects of theta-tDCS on brain electric activity and memory consolidation are strongly dependent upon ongoing brain state.

## Materials and Methods

### Subjects

Subjects were healthy students (medical or engineering) and university employees of comparable education level. All were native German speakers, nonsmoking, and medication-free at the time of the experiments. The exclusion criteria were based on presence or history of epilepsy, paroxysms, and cognitive impairments, mental, hormonal, metabolic or circulatory disorders. Subjects who reported sleep disturbances or an irregular sleep-wake cycle were not included. Subjects were first adapted to the experimental setup by spending one adaptation night in the sleep laboratory, and subjects with an abnormal sleep pattern on this adaptation night were excluded. The experimental protocol was approved by the ethics committee of the University of Lübeck, and each subject had given informed written consent prior to participating.

### Experimental design and procedures

Two separate experiments were conducted in which theta-tDCS (see below) and sham stimulation, respectively, were applied either during the first stable portion of NonREM sleep (NonREM Experiment) or stable REM sleep (REM Experiment). The NonREM Experiment involved 25 participants (14 women) with a mean age of 23.9 yr (age range 18–35 yr). Sixteen subjects (8 women) with a mean age of 24.3 yr (age range 21.3–30.6 yr) participated in the REM Experiment.

**Figure 1:**
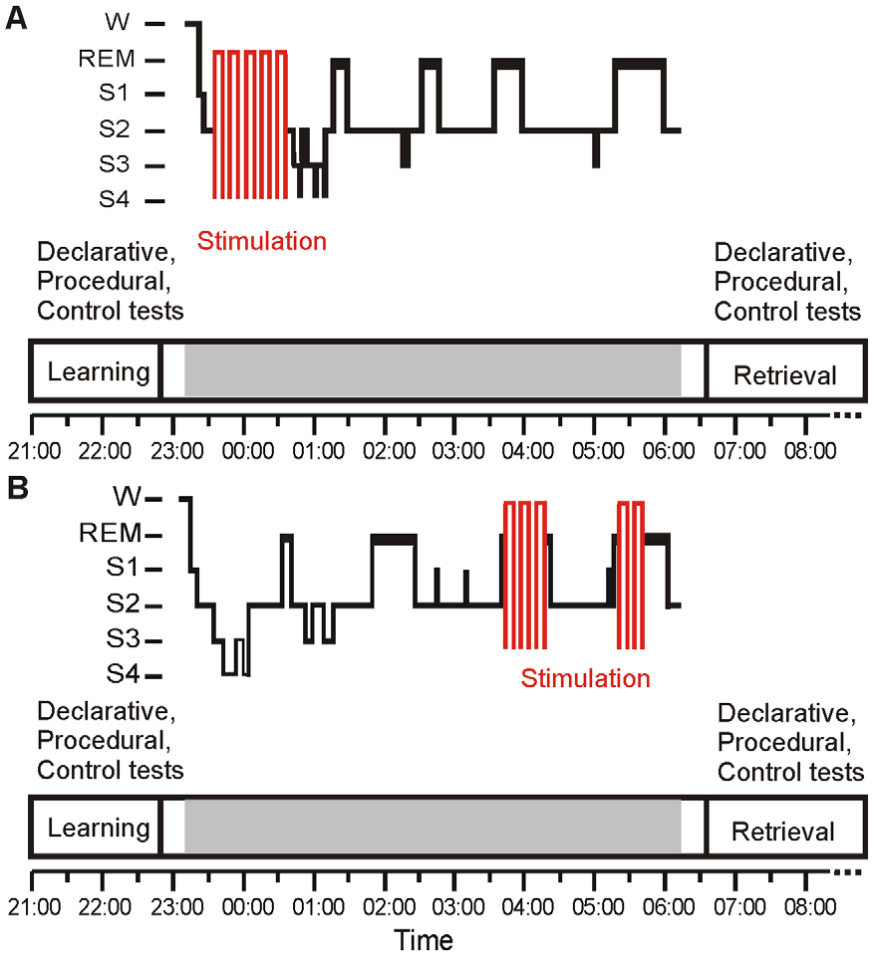
Time line of the two experiments. (A) Theta-tDCS during NonREM sleep (NonREM Experiment) and (B) during REM sleep (REM Experiment). Respective upper panels show representative individual sleep profiles (W: wake, REM: REM sleep, S1–S4: NonREM sleep stages 1–4) and periods of stimulation. Lower parts indicate Learning and Retrieval periods for memory testing on declarative and procedural tasks. The horizontal gray bar indicates lights out.

In the NonREM Experiment stimulation was turned on after the subjects had attained at least 4 min of stable NonREM sleep for the first time after sleep onset, that is a time when sleep is expected to progress into slow wave sleep (SWS). In the REM Experiment stimulation began at least 4 min after subjects had entered REM sleep after 3:00 am. Because in the latter experiment it was difficult to predict the duration of the REM sleep periods, stimulation often could not be readily applied during a single REM sleep period but frequently took place in 2 consecutive REM sleep periods upon their appearance. In the sham conditions, procedures were identical to those in the theta-tDCS conditions except that the stimulator remained off during sleep. The time course of both experiments is schematized in Figure 1.

For both experiments subjects arrived at the laboratory at 19:00 h. Following preparation for stimulation, EEG and polysomnographic recordings, subjects performed declarative and procedural memory tasks (see below) between 21:00 and 22:30 h (Learning period). The order of tasks was balanced across subjects. At 23:00 h subjects went to bed and recordings began. Awakening occurred as of 06:30 h when subjects were in light NonREM sleep (stages 1 or 2). About 30 min after awakening (07:00 h), recall of memories was examined (Retrieval period). Psychometric tests and control tests of cognitive functions were given before learning in the evening and/or before retrieval in the morning. Theta-tDCS and sham stimulation conditions for an individual subject were separated by an interval of at least 10 days and balanced in order across subjects. Findings from a small subset of the present data for the NonREM Experiment were described in Marshall et al. (2006).

### Theta-tDCS - 5 Hz oscillating anodal transcranial direct current stimulation

The current intensity of the sinusoidal stimulation fluctuated between 0 and 260 µA. Anodal theta-tDCS was induced by a battery driven constant current stimulator via stimulation electrodes (8 mm diameter/0.502 cm^2^) applied bilaterally at fronto-lateral locations, i.e., F3 and F4 of the international 10–20 system, with an ipsilateral reference electrodes placed at each mastoid. The bilaterally synchronized constant current was regulated independently for both circuits. At application electrode resistance was always below 2 kOhm. The estimated maximal current density at each of the two stimulating electrodes during the current peak corresponded to 0.517 mA/cm^2^. Post-experimental debriefing confirmed that stimulation was not felt by the subjects. The stimulation period consisted of five 5-min epochs of stimulation separated by 1-min stimulation-free intervals.

### EEG recordings

Both experiments recorded EEG with Ag-AgCl electrodes at Fz, C3, Cz, C4, P3, Pz, and P4, referenced to the nose, vertical and horizontal EOG, and submental EMG. The ground electrode was positioned on the forehead at Fpz. Electrode resistance was below 5 kOhm. Signals were recorded using Brain Vision Recorder (Brain Products GmbH, Gilching, Germany). On-line digital high and low pass filters were set at 0.08 and 45 Hz for the EEG and electrococulogram (EOG) and at 0.08 and 90 Hz for the electromyogram (EMG), with slopes of 24 dB/Oct for the high pass filters and 12 dB/Oct for the low pass. Signals were sampled at 200 Hz and amplified in a range of +/−3.2768 mV at a resolution of 0.1 µV. For the REM Experiment the high frequency cut off for EEG signals was increased to 150 Hz (500 Hz sampling rate) and the amplitude range increased to +/−16.384 mV (0.5 µV resolution). Thus, before spectral power analyses (see below) data of the REM Experiment were preprocessed, i.e., low pass filtered at 45 Hz with 12 dB/Oct and down sampled to 200 Hz by means of a spline interpolation method (Brain Vision Analyzer, Version 1.05, Brain Products).

### Analyses of sleep stages and EEG power

Two types of analyses were performed off-line. First, sleep structure was determined visually based on standard polysomnographic criteria [20], and second, the EEG power was calculated by means of Fast Fourier Transformations (FFT).

For the total sleep period every 30-s epoch was scored as wake, NonREM sleep stage 1, 2, 3, 4, REM sleep or movement time. Slow wave sleep (SWS) was determined as the sum of time spent in sleep stages 3 and 4. Latencies to SWS and REM sleep refer to time from the sleep onset, defined by the first occurrence of sleep sage 1 followed by sleep stage 2.

Intervals during acute stimulation were not scored due to the excessive signal artifacts. In the sham stimulation sessions, corresponding intervals (starting after the presence of eight consecutive 30-s epochs of stage 2 or deeper NonREM sleep or after the presence of 8 consecutive 30-s epochs of REM sleep, respectively) were also not scored. Scoring for the 1-min stimulation-free periods (between the 5-min blocks of acute stimulation) was performed for succeeding 10-s epochs. Time spent in the different sleep stages in the course of stimulation was thus determined for the 1-min stimulation-free periods and the corresponding 1-min period of the sham stimulation condition.

In addition, sleep stage classification based on 30-s epochs was conducted for a 60-min interval following the end of the stimulation period. For the REM Experiment sleep stage analyses were additionally performed separately for the first and second half of the night.

In a second, more fine-grained analysis, the immediate effect of stimulation on EEG power was investigated. Analyses were conducted using Brain Vision Analyzer (Version 1.05, Brain Products). Six EEG intervals were selected for the analyses: a 1-min period immediately before theta-tDCS (baseline) and the five 1-min stimulation-free periods immediately after each 5-min block of stimulation (including the interval after the last stimulation). Four to six non-overlapping blocks of artifact-free EEG with 2,048 data points each (≈10.2 s) were used for every 1-min stimulation-free interval and corresponding intervals of sham stimulation. On every 2,048-point block of EEG data, a Hanning window (20%) was applied before calculating the power spectra using FFT (resolution≈0.097 Hz). Individual mean power spectra across all blocks of a stimulation-free interval were calculated and subjected to a three-point moving average. Mean power was calculated for the following frequency bands: slow oscillations (0.5–1 Hz), delta (1–4 Hz), theta (4–8 Hz), slow spindle (8–12 Hz), fast spindle (12–15 Hz), beta (15–25 Hz), and gamma (25–45 Hz).

In the sham conditions, FFT was conducted on EEG intervals corresponding to the time periods used in the respective theta-tDCS conditions. In order to investigate possible EMG contributions to the EEG power in the frequencies above 15 Hz, the corresponding EMG signal was also subjected to FFT. For statistical analyses, the individual mean power within the 1-min baseline interval immediately preceding stimulation and sham-stimulation onset was subtracted from the mean power during stimulation-free intervals.

To characterize longer lasting after effects in the EEG after cessation of theta-tDCS, in the NonREM Experiment mean EEG and EMG power was calculated for two consecutive 30-min intervals following the end of the 5th stimulation or sham-stimulation, as described above. Epochs containing artifacts, sleep stages different from stage 2 NonREM sleep or SWS were excluded from the analyses. This resulted into 120–125 EEG segments for each 30-min period. For stimulation during REM sleep, long lasting after effects of stimulation were not explored because of the variable temporal distribution of the REM sleep periods used for stimulation and the quite heterogeneous distribution of sleep stage following stimulation among the subjects.

### Memory tasks

One declarative (word paired-associates) and two procedural memory tasks (finger sequence tapping, mirror-tracing) were used in the two experiments. For all tasks, parallel versions (A, B) were used in the subject's two experimental sessions.

In the word paired-associate learning task a list of forty-six semantically related pairs of German nouns (e.g., bird-claw) were presented on a PC monitor at a rate of 1/5 s and an interstimulus interval of 100 ms. Also, four dummy pairs of words shown in sequence at the beginning and end of each list served to buffer primacy and recency effects, respectively. At learning, before the retention period, presentation of the list was immediately followed by a cued recall, i.e., the subject was to respond by naming the second word on presentation of the first (cue) word of each pair, whereby the 46 stimulus words of the word list appeared on the screen in a different order than during the foregoing presentation. The subject had unlimited time to recall the appropriate response word. If a minimum of 60% correct responses was not obtained on a run, word-pairs were presented again in a newly randomized order (to prevent serial learning) and the cued recall was repeated. At retrieval testing in the morning cue words were again displayed in a newly randomized order and the subject was required to recall the appropriate response words. Overnight memory retention is represented by the difference in the number of words recalled at morning retrieval minus the number of words reproduced correctly at evening immediate recall.

In the finger sequence tapping task which was adopted from Walker and colleagues [21] subjects were required to repeatedly finger-tap with the non-dominant left hand a five-element sequence presented on a computer monitor as fast and accurately as possible on a key board. The two sequences used were “4-2-3-1-4” and “4-1-3-2-4.” The training period before sleep consisted of twelve 30-s trials with 30-s breaks between the trials. Retrieval testing at morning consisted of a practice run followed by three 30-s test trials. A working memory component of the task was excluded by continuous presentation of the sequence on the screen. No feedback was given on pressing keys. Each 30-s interval was scored for the number of correctly completed sequences (speed) and the number of errors made (error rate). Performance at learning and retrieval testing, respectively, was defined by averaged scores across the final three 30-s trials during the learning period, and across the three test trials of the retrieval period. Retention performance was defined by the difference between performance at retrieval testing and at learning.

In the mirror tracing task [12] the subject had to trace as fast and as accurately as possible line-drawn meaningless figures while these figures (with 26 to 27 angles and curved corners) and the subject's hand movements were visible only through a mirror. Subjects traced each figure with an electronic stylus starting and ending at the same point. Drawing speed and error rate were registered. An error consisted of moving the stylus off the line of the figure. At learning, subjects first performed practice runs with a star-like figure until draw time was <1 min and number of errors made <12 (the learning criteria), and then continued with 6 runs on the test figure. At retrieval testing, after one practice run, performance on 6 runs on the test figure was examined. On each occasion, the total time to trace the figure, and the number of errors were measured and averaged across the 6 test runs. Retention was defined by the difference in performance on the test figure at retrieval testing minus performance during the learning period.

### Psychometric and cognitive control tests

Subjective mood, motivation and feelings of activation and tiredness were assessed by the positive and negative affect scale (PANAS) [22], an adjective checklist (EWL) [23] and the Stanford Sleepiness Scale. In the morning, subsequent to recall testing a word fluency task served to assess the capability to retrieve information from long term memory [24]. Working memory function was measured using the Digit Span test of the Wechsler Adult Intelligence Scale [25].

### Statistical analyses

Statistical analyses was performed with SPSS version 15, for Windows and relied basically on analyses of variance (ANOVA) with post-hoc pairwise testing to specify significant main and interaction effects. Prior to ANOVA, normal distribution of the data was assured using the Kolmogorov-Smirnov test. All ANOVA included a repeated measures factor Stimulation (theta-tDCS vs sham). For most behavioral measurements an additional repeated measures factor Time was introduced with 2 levels (evening, morning), and for analyses of EEG data across the 25-min stimulation period levels were represented by the five stimulation-free 1-min epochs subsequent to the 5-min periods of actual stimulation. The factor Lead (Fz, C3, Cz, C4, P3, Pz, P4) was also used. EEG power during the stimulation-free 1-min epochs and during the 60-min period after stimulation was analyzed. Statistical results on data conducted after individual subtraction of the baseline value during the 1-min period before stimulation onset did not differ essentially from those conducted using baseline value as an additional level of the Time factor. EEG power was subjected to ANOVA separately for the different frequency bands of interest. To avoid Type I errors due to multiple comparisons, for the main effect of Stimulation a Bonferroni corrected P-value<0.007 was adopted.

## Results

### Theta-tDCS during NonREM sleep lightened sleep acutely

We first investigated immediate effects of theta-tDCS on polysomnographically determined sleep. Since the acute period of stimulation produces extreme disturbances in the EEG, analyses of acute effects were conducted on the five 1-minute stimulation-free epochs immediately subsequent to each 5-minute stimulation period. Theta-tDCS strongly reduced the mean time spent in SWS within the five stimulation-free periods (theta-tDCS 94.80±9.90 vs. sham 166.80±14.85 s, F_1,24_ = 13.08, P = 0.001) as well as the typical increment in SWS over time (F_4,96_ = 3.31, P = 0.02, for Stimulation×Time interaction). The lightening of sleep during this time was also reflected by an increased amount of stage 2 sleep (theta-tDCS 202.80±10.22 vs. sham 132.00±14.33 s, F_1,24_ = 12.64, P = 0.005), while stage 1 sleep was unaffected (see Table 1). Theta-tDCS also increased the latency to SWS onset on average by 2.66±1.28 min (F_1,24_ = 4.34, P<0.05; Table 2), reflecting thus the increment in SWS over time.

**Table 1:** Sleep during the five 1-min stimulation-free and the 60 min periods following theta-tDCS during NonREM sleep.

	Stimulation period [s]		First 30-min interval [%]		Second 30-min interval [%]	
	Sham	Theta-tDCS	Sham	Theta-tDCS	Sham	Theta-tDCS
Awake	0.00±0.0	0.4±0.4	0.13±0.92	0.13±0.92	0.40±0.24	0.40±0.19
S1	1.20±1.2	2.0±1.2	0.20±0.14	0.13±0.38	1.06±0.09	0.60±0.30
S2	132.0±14.3	202.8±10. 2^2^	37.93±1.47	29.3±2.03^1^	54.53±2.00	56.53±2.60
S3	146.0±12.6	80.4±8.6^2^	46.80±1.45	51.86±1.81^1^	26.40±1.88	26.46±2.33
S4	20.8±4.0	14.4±3.4	14.86±0.60	18.40±0.76^2^	7.53±1.64	7.53±1.63
SWS	166.8±14.9	94.8±9.9^2^	61.66±1.50	70.26±2.05^1^	33.93±3.16	34.00±3.26
REMS	0.0±0.0	0.0±0.0	0.00±0.00	0.00±0.00	9.53±2.52	8.00±2.81

Sleep stage scoring for the five 1-minute stimulation-free periods is based on 10-s with mean ± SEM time in the different sleep stages given in seconds. Sleep stage scoring for the 60 minutes following theta-tDCS is based on 30-s epochs with mean ± SEM time in the different sleep stages given as percentage of 30 min. S1–S4, sleep stages 1–4; SWS, Slow wave sleep; REMS, rapid eye movement sleep.

1 P<0.001;

2 P<0.01, for pairwise comparisons between the effects of theta-tDCS and sham stimulation.

**Table 2:** Sleep during the whole night.

	NonREM Experiment		REM Experiment	
	Sham	Theta-tDCS	Sham	Theta-tDCS
TST (min)	432.68±3.19	432.96±3.67	431.75±5.00	432.90±5.54
SO latency (min)	15.72±1.37	15.78±1.83	16.65±4.48	16.73±4.45
SWS latency (min)	17.28±1.43	19.94±1.00*	17.59±2.10	17.15±1.44
REM latency (min)	97.18±2.90	96.80±2.35	99.75±8.44	98.03±9.11
Wake (%)	1.36±0.40	1.32±0.32	1.70±0.71	0.61±0.21
Stage 1 (%)	3.85±0.55	3.89±0.71	4.68±0.65	4.55±1.00
Stage 2 (%)	56.85±0.91	56.97±1.04	55.80±1.53	55.15±1.57
Stage 3 (%)	8.81±0.53	8.64±0.54	9.07±0.53	10.21±0.43
Stage 4 (%)	3.97±0.51	4.15±0.53	5.11±1.01	5.16±1.13
SWS (%)	12.78±0.89	12.79±0.81	14.13±1.17	15.37±1.23
REM sleep (%)	18.41±0.51	18.46±0.54	17.24±1.40	17.80±1.42

Sleep stage scoring for both NonREM sleep and REM sleep experiments is based on 30-s epochs and mean ± SEM time in the different sleep stages is given as percentage of total sleep time (TST). SO, Sleep onset; SWS, Slow wave sleep. Latency (in min) of sleep onset is calculated with reference to lights off (23.00 h); latencies to SWS and REM sleep are calculated with reference to sleep onset.

*P<0.05 for longer SWS latency in the theta-tDCS than sham condition.

Theta-tDCS lightened sleep only within the five stimulation-free epochs. Analyses of the 1-hour interval following theta-tDCS, a period analyzed to characterize prolonged after-effects of stimulation, revealed a distinct rebound of SWS that was restricted to the first 30-min period of this interval (tDCS 21.08±0.62, sham 18.50±0.45 min; P<0.001, F_1,24_ = 74.07, P<0.001, for Stimulation×Time interaction; Table 1). Simultaneously, stage 2 NonREM sleep was reduced during this first 30-min interval after stimulation (theta-tDCS 8.78±0.61, sham 11.38±0.44 min, P<001, F_1,24_ = 68.23, P<0.001 for Stimulation×Time interaction). Stimulation did not alter sleep architecture as assessed by full-night polysomnography (Table 2).

### Theta-tDCS during NonREM sleep acutely decreases slow oscillation and spindle power

**Figure 2:**
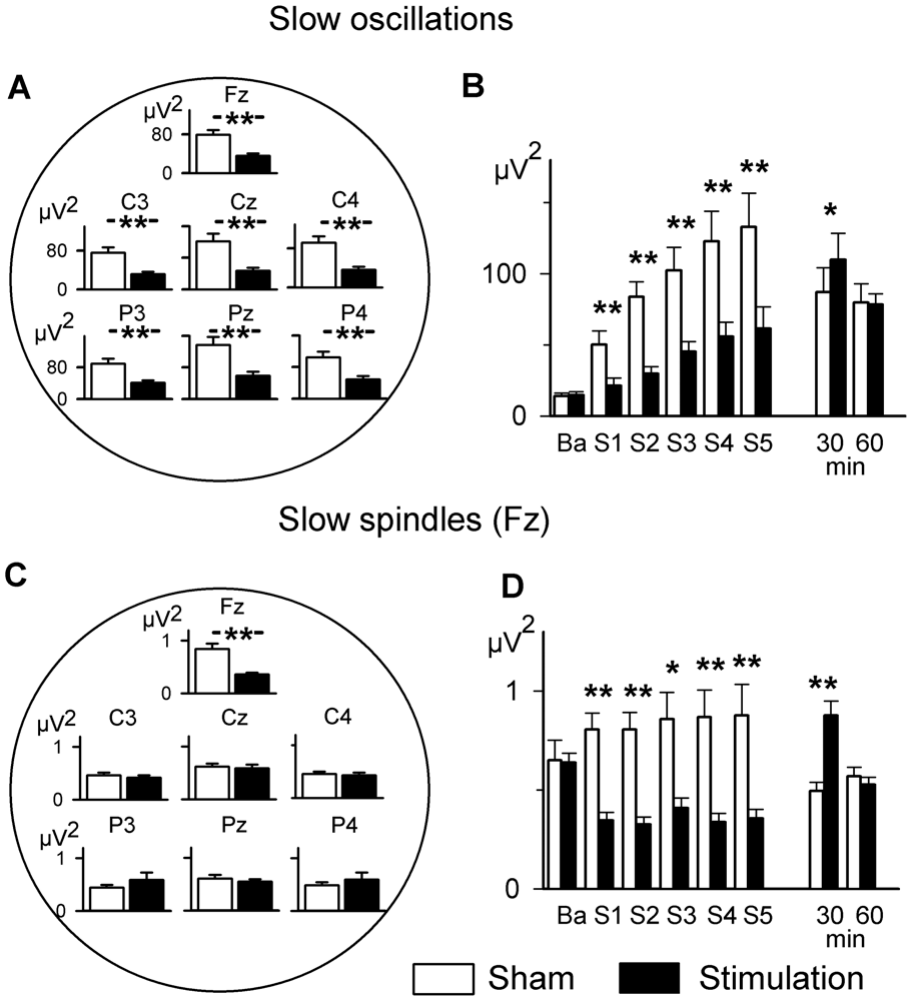
Topographical distribution and temporal pattern of EEG spectral power during NonREM sleep. (A and C) Topographical distribution of EEG power (± SEM) averaged across the 1-min stimulation-free intervals in the slow oscillation (0.4–1.2 Hz) (A), and slow spindle (8–12 Hz) frequency bands (C) after the five 5-min intervals of theta-tDCS or sham stimulation. (B and D) Time course of EEG spectral power for the five 1-min stimulation-free periods (S1–S5) immediately succeeding stimulation and for 0–30 and 30–60 min after termination of stimulation in the slow oscillation band (B), and slow spindle frequency band at Fz (D). ‘Ba’ refers to baseline activity prior to stimulation. **, P<0.01; *, P<0.05; for comparisons between the theta-tDCS and sham stimulation conditions by t-test (n = 25).

To better understand the actions of theta-tDCS on NonREM sleep we analyzed rhythmic EEG activity in the frequency bands of interest. In parallel with reduced SWS, we found a distinct decrease in EEG power in the slow oscillation (0.5–1 Hz; F_1,24_ = 33.08, P<0.001) and delta (1–4 Hz; F_1,24_ = 48.33, P<0.001) frequency bands (Fig. 2A, B; changes in slow wave activity defined by the joint 0.5–4 Hz range generally reflected changes in the slow oscillation frequency band and are not reported here separately). The theta-tDCS induced reduction in slow oscillation power was particularly pronounced towards the end of the stimulation period (F_5,120_>9.74, P<0.001, for Stimulation×Time) and for the frontal and midline central and parietal leads (F_6,144_>10.94, P<0.001; Stimulation×Lead). Importantly, theta-tDCS also reduced EEG power in the 8–12 Hz frequency band, but only at Fz (F_6,144_ = 8.93, P = 0.002; for Stimulation×Lead interaction; F_1,24_ = 20.79, P<0.001 for the effect of Stimulation at Fz, P>0.22, for all other leads; Fig. 2C, D) with this frequency band covering the slow frontal spindle activity (see Mölle and colleagues 2004 [26], Fig. 3 of SI for illustration of the frontal peak in 8–12 spectral power characterizing slow spindle activity during SWS). EEG spectral power in the fast spindle band (12–15 Hz) was not affected by theta-tDCS (P>0.9).

**Figure 3:**
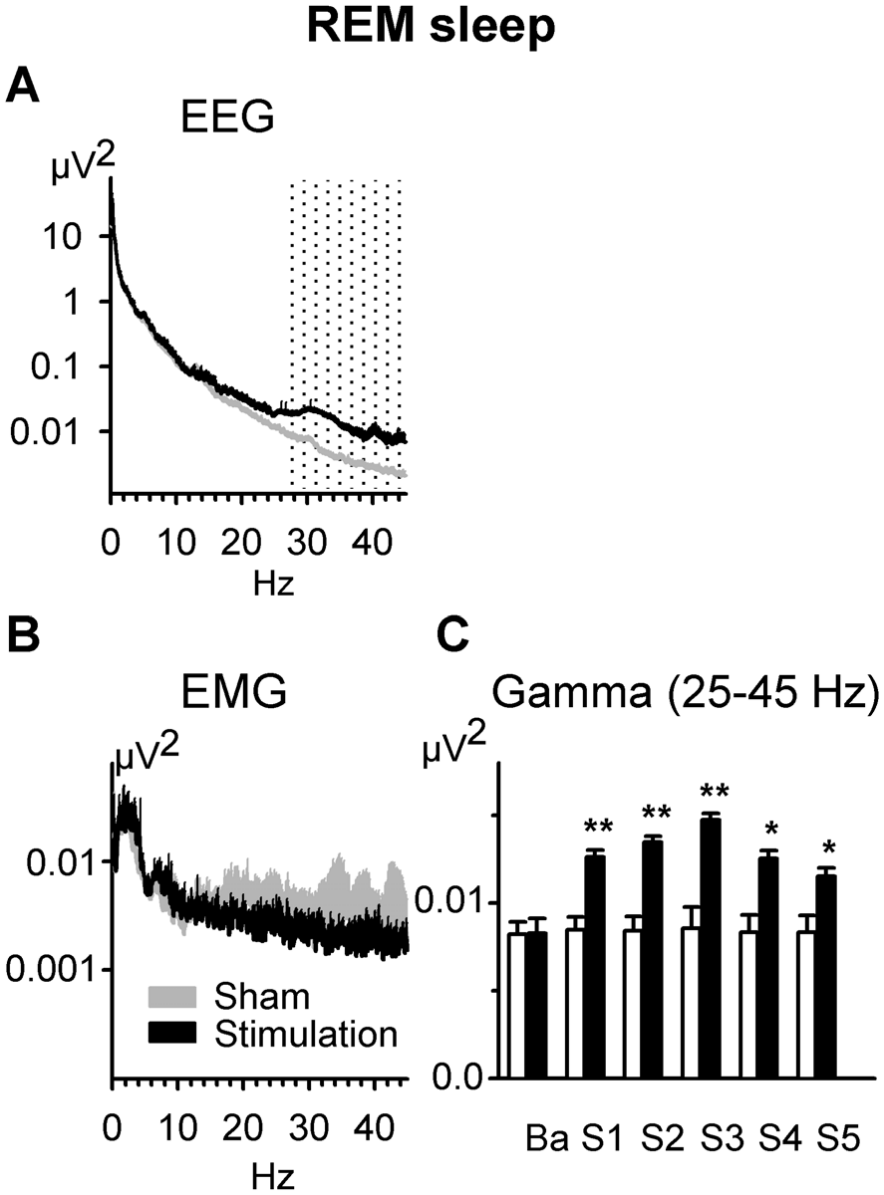
Changes in EEG spectral power induced by theta-tDCS during REM sleep. Spectral power for theta-tDCS (black lines) and sham stimulation (gray lines) averaged across all EEG locations (A) and for submental electromyographic (EMG) activity (B). Time course of gamma band activity during the five 1-min stimulation-free periods immediately succeeding the stimulation intervals (S1–S5), averaged across all electrode sites for theta-tDCS (black bars) and sham stimulation (empty bars) (C). ‘Ba’ refers to baseline activity prior to stimulation. **, P<0.01; *, P<0.05; for comparisons between the theta-tDCS and sham stimulation conditions by t-test (n = 16).

Similar to SWS, slow oscillation power also revealed a strong rebound during the first 30-min interval following cessation of theta-tDCS (F_1,24_ = 15.96, P<0.001), with no measurable difference between conditions during the second 30-min interval (P>0.8; F_1,24_ = 4.54, P = 0.04; for Stimulation×Time, Fig. 2B). Slow spindle activity (8–12 Hz) at the frontal (Fz) location also showed a rebound restricted to the first 30-min period after stimulation (F_1,24_ = 48.65, P<0.001; F_6,144_ = 14.48, P<0.001, for Stimulation×Time×Lead, Fig. 2D). No significant differences between conditions in any other EEG frequency band during the first or second 30-min intervals were found (P>0.13 for all relevant comparisons).

### Theta-tDCS during REM sleep increases EEG gamma band activity

When applied during REM sleep, theta-tDCS did not change late sleep architecture (Table 2) nor sleep within the five stimulation-free periods. The latter contained almost entirely REM sleep (Stimulation: 296.25±1.54 s vs. Sham 295.63±1.57 s, P>0.8).

Power spectral analyses of the EEG signal during the stimulation-free periods did not reveal any pronounced changes in the EEG frequency bands below 15 Hz (P>0.34; for all relevant comparisons). However, theta-tDCS strongly increased gamma (25–45 Hz) EEG power (F_1,15_ = 19. 59, P<0.001) with this effect equally distributed across all electrode locations (P>0.9 for Stimulation×Lead interaction). The effect was most pronounced during the third and less expressed during the fourth and the fifth stimulation-free periods (F_5,75_ = 7.20, P<0.001 for Stimulation×Time; Fig. 3). Activity in the neighboring beta frequency band also tended to be affected by theta-tDCS in the third stimulation-free period (P = 0.053, for pair wise contrast; F_5,75_ = 3.88, P<0.05, for Stimulation×Time). Because submental electromygraphic (EMG) activity within the beta and gamma frequency ranges during the stimulation-free periods showed no difference between conditions (P>0.18), a contamination of gamma band activity by muscle activity can be excluded.

### Theta tDCS during NonREM sleep decreases memory performance

**Figure 4:**
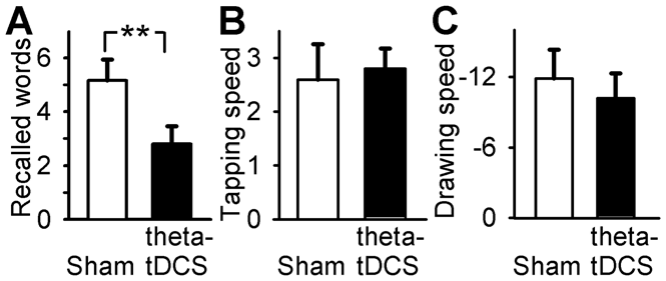
Theta-tDCS during NonREM sleep impairs declarative memory consolidation. (A) Retention performance on the declarative memory task (word paired-associates) across nocturnal sleep for the sham control and theta-tDCS conditions. Performance is expressed as difference between the number of correct words reported in a cued recall test at retrieval testing after sleep and in the end of learning before sleep. The list contained 46 word-pairs (**P<0.01). Performance across the retention interval on (B) the procedural finger sequence tapping task expressed as the difference in the number of correctly tapped sequences per 30 s between retrieval testing and learning, and on (C) the procedural mirror drawing task expressed as the corresponding difference in drawing time. Data are the means ± SEM. Times of performance testing within the experiments are depicted in Fig. 1.

We found that application of theta-tDCS during NonREM sleep strongly impaired consolidation of the declarative word pairs. Whereas in the sham-stimulation condition subjects recalled 5.16±0.78 more word pairs after sleep than at the end of learning prior to sleep, retention performance after theta-tDCS dropped to 2.80±0.65 word pairs (F_1,24_ = 9.087, p = 0.006, Fig. 4). Neither consolidation of procedural finger sequence tapping nor mirror-tracing skills were affected by theta-tDCS (P>0.30 for the main effect of Stimulation and Stimulation×Time interaction, Table 3, Fig. 4).

**Table 3:** Performance at learning and retention.

Learning		Retention	
Sham	Theta-tDCS	Sham	Theta-tDCS
Word paired-associates (recalled words) – NonREM			
35.32±1.13	36.96±0.99	5.16±0.78	2.80±0.65**
Tapping speed (total number of tapped sequences) – NonREM			
19.04±1.17	18.54±1.11	2.59±0.66	2.79±0.38
Tapping accuracy (number of errors) – NonREM			
1.46±0.24	1.60±0.46	−0.10±0.39	−0.15±0.40
Mirror-tracing speed (drawing time in sec) – NonREM			
80.13±4.32	80.30±4.32	−11.87±2.44	−10.20±2.09
Mirror-tracing errors (number of errors) – NonREM			
24.82±2.49	25.56±3.00	−7.85±1.73	−10.93±2.21
Word paired-associates (recalled word) – REM			
38.75±1.99	37.00±1.19	0.50±0.65	0.81±0.87
Tapping speed (total number of sequences) – REM			
19.46±0.93	21.71±1.41	2.25±0.70	3.33±0.65
Tapping accuracy (number of errors) – REM			
1.60±0.20	1.43±0.28	−0.31±0.39	−0.43±0.58
Mirror-tracing speed (drawing time in sec) – REM			
63.35±4.54	63.52±4.73	−15.90±3.55	−11.63±2.69
Mirror-tracing errors (number of errors) – REM			
22.75±1.80	22.74±1.71	−6.53±1.23	−6.23±0.90

Mean ± SEM values are given for performance at learning and retention performance on the declarative word paired-associate task and the two procedural tasks, i.e., finger sequence tapping and mirror tracing. Retention is defined by the difference in performance at retrieval testing (morning after sleep) minus performance at immediate recall at learning (evening before sleep). No significant differences between the theta-tDCS and sham conditions were found for performance at learning.

**P<0.01 for retention with sham vs. theta-tDCS.

For theta-tDCS during both NonREM and REM Experiments there were no differences in performance at learning before sleep on either the declarative or procedural tasks (Table 3). In general better task performance was observed at morning recall as compared to evening learning (P<0.01), with the exception of the number of errors on finger-sequence tapping and the recall of word-pair associates which failed to significantly differ across sleep, in the NonREM and REM Experiments, respectively (P>0.34).

### Performance on psychometric and cognitive control tests

Results on psychometric and cognitive control tests are given in Table 4. Significant effects were found only for REM sleep. Here theta-tDCS was associated with a relative increase in the PANAS negative score as compared to sham (P<0.02). Also, the lower number or words produced by subjects in the morning after theta-tDCS (P = 0.03), suggests an impairment in verbal word fluency at this time (Table 4). The slight reduction in mean working memory performance as indicated by the digit span task did not obtain significance (P = 0.06). The absence on any further significant differences between stimulation and sham on any other control measure of the NonREM or REM Experiments excludes that the reduced retention of word pairs observed for theta-tDCS during NonREM sleep was confounded by any difference in stimulus encoding, recall ability or other non-specific effects of arousal or mood.

**Table 4:** Performance on psychometric and cognitive control tests.

NonREM Experiment		REM Experiment	
Sham	Theta-tDCS	Sham	Theta-tDCS
PANAS, positive score (learning)			
2.70±0.10	2.82±0.09	2.67±0.12	2.74±0.15
PANAS, positive score (retrieval)			
3.05±0.12	2.97±0.12	3.04±0.14	2.96±0.17
PANAS, negative score (learning)			
1.10±0.03	1.12±0.04	1.13±0.05	1.11±0.05
PANAS, negative score (retrieval)			
1.21±0.15	1.14±0.07	1.05±0.03	1.20±0.10†
Word fluency (retrieval)			
18.38±0.71	17.74±0.64	18.50±0.66	17.00±0.46*
Digit span (retrieval)			
9.42±0.32	8.78±0.35	8.34±1.33	8.12±1.47

Mean ± SEM values for the assessment of mood (by the PANAS), retrieval ability from long-term memory (by the Word fluency test) and working memory (by the Digit Span test in the NonREM Experiment and the REM Experiment. All assessments were conducted at retrieval testing (in the morning after sleep). The PANAS was given additionally at learning (in the evening before sleep).

*P<0.05 for pairwise comparisons between the effects of theta-tDCS and sham stimulation.

† P<0.05 for the interaction Stimulation×Time (learning, retrieval).

## Discussion

### Theta-tDCS during NonREM sleep acutely decreases SWS, slow oscillation and spindle power

The finding in the present study of a conjoint suppression of slow oscillations and slow frontal spindle activity during tDCS oscillating at a 5-Hz theta frequency is remarkably complementary to the finding that tDCS oscillating at ∼0.75 Hz (slow oscillation-tDCS) enhanced endogenous slow oscillation and slow frontal spindle activity [2]. Together, with the finding of enhanced theta activity by slow oscillation-tDCS during wakefulness [5] these data support the idea that the effects of oscillatory-tDCS are strongly dependent on stimulation frequency and brain state. Moreover, the complementary actions of slow oscillation-tDCS enhancing endogenous theta rhythm (during waking) and theta-tDCS suppressing the endogenous slow oscillation rhythm (during sleep), as reported here, strongly argue for a coupling mechanism between the cortical networks underlying both theta and slow oscillation EEG rhythms. Increased theta activity during extended periods of wakefulness is indeed positively correlated with pronounced slow oscillatory activity, particularly over frontocortical areas [27], [28].

It is well documented that SWS and associated slow oscillatory activity are homeostatic in nature becoming enhanced subsequent to total sleep deprivation or selective SWS deprivation [29]–[31]. Hence the finding that the suppression of slow oscillatory activity as induced by the relatively short period of theta-tDCS (25 minutes) was followed by a rebound in activity, indicating an impact on this homeostatic mechanism, is a further example of the functional significance of transcranial electric stimulation. Moreover, since applied transcranial electric currents exert initial effects presumably by an immediate modulation of electric fields on cortical networks [3], [4], [6], [7] the occurrence of a rebound already within the first half hour following stimulation support the existence of a fast-acting cortical mechanism underlying homeostatic sleep regulation. Whether indeed sleep-regulatory substances are released cortically by the polarizing effect of stimulation, or by the stimulation-induced EEG rhythms in a fashion similar to the activity-dependent release of sleep-regulatory substances, has yet to be investigated [32], [33].

Interestingly, the suppression of slow oscillation activity during theta-tDCS and its subsequent rebound were accompanied by parallel changes in slow frontal spindle activity whereas fast spindle activity which shows a more widespread centro-parietal distribution remained unaffected. Previous studies have likewise observed rebound activity after periods of sleep deprivation for slow frontal but not for fast centro-parietal spindles [29], [31], [34], [35]. Both kinds of spindles are grouped by the neocortical slow oscillation, however at different phases, with the slow frontal spindles occurring preferentially at the transition into the hyperpolarizing phase of the slow oscillations and fast spindles occurring typically in the depolarizing phase [36]; Mölle, unpublished results). Together these findings corroborate the existence of two types of spindles with different underlying generating mechanisms. Although there is still no complete consensus on the sources in particular of slow spindle activity (e.g., [37]), a high resolution EEG study concluded that slow spindles result from cortico-cortical activation following spindle initiation, whereas fast spindles reflect thalamo-cortical activation [38]. A dominant frontal neocortical source could well explain the susceptibility to theta-tDCS that we found here selectively for slow, but not for fast spindle activity.

### Theta-tDCS during REM sleep increases EEG gamma band activity

Although in human REM sleep a pronounced spectral peak of theta power within the surface EEG is typically absent, anterior cortical theta generators and enhanced frontal theta activity have been discerned [18], [19], [28]. The present enhancement of broadband gamma EEG activity by theta-tDCS indicates that theta-tDCS was indeed effective, apparently boosting a rhythm inherent to the present state, but not maximally expressed at the time of stimulation. Increased magnetoencephalographic, intracranial, and also EEG gamma band activity have been detected in REM sleep as compared to SWS [39]–[41], although not consistently [42]. Although gamma oscillations are thought to transiently link local cell assemblies processing closely related information [43], the widespread increase in cortical gamma band activity with theta-tDCS supports a functional coupling between these rhythms, with the theta rhythm presumably serving to synchronize gamma band activity occurring at distant regions and/or increase its coherence. Enhanced cross-frequency coupling between theta and gamma oscillations in the human neocortex is found for memory formation processes during waking [44]–[46]. Moreover, modulation of gamma power by weak electric fields in a hippocampal slice exhibiting theta oscillations was recently shown and modeled computationally based on the induced effects on firing rate and spike timing [47].

### Theta tDCS during NonREM sleep decreases memory performance

Consolidation of the hippocampus-dependent word paired-associate learning task, measured as the difference between performance at learning before sleep and at retrieval testing after sleep, has consistently been shown to benefit from SWS and associated slow oscillation activity dominant during early nocturnal sleep [2], [10], [11], [26], [47], [48]. Memories for procedural skills such as finger sequence tapping and mirror-tracing were often shown to benefit more from REM sleep dominating late nocturnal sleep [12], [21], [49]–[51], although there is also evidence that EEG theta activity during REM sleep is increased following learning of declarative word-pairs [52].

Previously, slow oscillation-tDCS (∼0.75 Hz) led to a robust enhancement in the consolidation of word-pair memories together with an increase in endogenous slow oscillatory and frontal slow spindle activity [2]. Here, theta-tDCS produced exactly the opposite results, i.e., an impaired consolidation of word pair memories in the presence of reduced slow oscillation activity and frontal slow spindle activity. This strengthens previous findings in which no effect on memory consolidation was found after theta-tDCS with a small sample of 5 subjects [2]. In combination, these observations further corroborate the concept of slow oscillations as a mechanism for coordinating putative sleep-associated processes of consolidation.

The sleep slow oscillation may provide a temporal frame for the transfer of newly encoded memories from hippocampal to neocortical sites by synchronizing thalamic spindle and hippocampal ripple activity, presumably involving also a coordination between slow oscillatory rhythmic network activities of the neocortex and hippocampus [11], [53]–[55]. Notably, theta-tDCS induced a decrease in retention performance in spite of the observed rebound of both SWS and SWA as well as spindle activity that occurred during the 30 min period following the stimulation. Hence, suggesting that the mechanisms underpinning consolidation of the hippocampus-dependent memory take place mostly during the initial transition into nocturnal SWS.

The failure of theta-tDCS applied during REM sleep to affect consolidation of either procedural skills or declarative paired-associate words despite a global increase in gamma band activity indicates that brain electric activity associated with theta and gamma rhythms during REM sleep is less essential for memory consolidation, and may be effective only in conjunction with changes in memory representations induced during preceding NonREM sleep [56]–[58]. Recent research indicated that pharmacological suppression of REM sleep by antidepressant drugs did not impair consolidation of procedural or declarative memory [14], thus adding to growing evidence that REM sleep exerts a permissive rather than an immediate influence on memory consolidation. Indications in the present study for an influence of theta-tDCS during REM sleep on control tests are supportive of processes during REM affecting cortical associations and emotional tone of dreams and/or memories [59], [60].

Notably, since theta-tDCS during REM and NonREM sleep occurred during different times of night circadian influences, e.g., the circadian regulation of neuronal excitability, but also an effect or interaction with homeostatic sleep mechanisms on the reported EEG and/or cognitive effects may not be completely excluded [30], [61], [62]. We have however no reason to assume that the present results were biased by effects of circadian rhythm or sleep drive.

### Conclusion

The present findings underline the strong dependence of oscillatory-tDCS effects of comparable amplitude on ongoing network activity and brain-state [5], [51], [63], [64]. Furthermore, data indicate the efficacy of oscillatory-tDCS to enhance or suppress the expression of physiologically coupled EEG rhythms and associated processes such as memory consolidation, which makes this kind of stimulation a useful tool for systematic investigations of brain electric rhythms. Finally, since theta-tDCS at the applied amplitude is expected to induce fields similar in strength to those which occur endogenously [65], results can be taken as further support for the functional significance of endogenous electric fields in cortical network activity [6], [8].
